# Ferroptosis: A Specific Vulnerability of RAS-Driven Cancers?

**DOI:** 10.3389/fonc.2022.923915

**Published:** 2022-07-15

**Authors:** Cristina Andreani, Caterina Bartolacci, Pier Paolo Scaglioni

**Affiliations:** Department of Internal Medicine, University of Cincinnati College of Medicine, Cincinnati, OH, United States

**Keywords:** RAS-driven cancer, ferroptosis, CRISPR screening, cancer dependency map, cell death

## Abstract

Ferroptosis has emerged as a new type of programmed cell death that can be harnessed for cancer therapy. The concept of ferroptosis was for the first time proposed in in the early 2000s, as an iron-dependent mode of regulated cell death caused by unrestricted lipid peroxidation (LPO) and subsequent plasma membrane rupture. Since the discovery and characterization of ferroptosis, a wealth of research has improved our understanding of the main pathways regulating this process, leading to both the repurposing and the development of small molecules.

However, ferroptosis is still little understood and several aspects remain to be investigated. For instance, it is unclear whether specific oncogenes, cells of origin or tumor niches impose specific susceptibility/resistance to ferroptosis or if there are some ferroptosis-related genes that may be used as *bona fide* pan-cancer targetable dependencies. In this context, even though RAS-driven cancer cell lines seemed to be selectively sensitive to ferroptosis inducers, subsequent studies have questioned these results, indicating that in some cases mutant RAS is necessary, but not sufficient to induce ferroptosis. In this perspective, based on publicly available genomic screening data and the literature, we discuss the relationship between RAS-mutation and ferroptosis susceptibility in cancer.

## Introduction and Open Questions

Ferroptosis is a type of programmed-cell death characterized by iron- and reactive oxygen species (ROS)-dependent lipid peroxidation (LPO) (1, 2). It was initially found to be induced by small molecules identified in a screen for compounds able to selectively kill isogenic cancer cell lines carrying a mutant form of RAS, thus suggesting a connection between RAS oncogene and ferroptosis (1, 3–6).

Since then, many studies have continued linking RAS mutation/activation to oxidative stress and ferroptosis susceptibility (7–12).

However, several open questions remain:

1. Is ferroptosis an actionable dependency for cancer therapy independently of RAS mutation/activation?

2. Can RAS-mutation/activation predispose cancer cells to ferroptosis?

3. Do specific tumor microenvironments, niches or cells of origin predispose RAS-driven cancers to ferroptosis?

Giving a definite answer to these questions is certainly challenging and requires a brief overview of the ferroptosis pathway itself, extensively reviewed elsewhere (2, 13–15).

Without providing a comprehensive description of all the genes/proteins involved in ferroptosis, which goes beyond the scope of this perspective, what we can conclude is that, even though the outcome of ferroptosis consists of LPO and cell death, several different paths can lead to this deadly end. In this context, several studies have demonstrated that specific cancers are addicted to one or more of these pathways whereas being totally independent of others (16–19). Also, it is emerging that under stress (including cancer therapy, metastatic process, metabolic changes, redox imbalance) cancer plasticity, heterogeneity and adaptations might dictate a different path toward ferroptosis.

How are RAS mutations contributing to this scenario?

To our knowledge, our study done in mutant KRAS (KM) lung cancer (LC), represents the first evidence of a direct causal relationship between KM and sensitivity to ferroptosis *via* fatty acid synthase (FASN) inhibition in LC (11). In this case FASN is directly preventing LPO in KMLC cells by providing oxidation-resistant fatty acids, mainly saturated and monounsaturated fatty acids (SFA and MUFA). Yet, this study also unravels how the LC microenvironment, rich in polyunsaturated fatty acids (PUFA), is crucial to predispose cancer cells to ferroptosis, adding an additional layer of complexity (11). Similarly, lymph microenvironment has been found to protect metastasizing melanoma cells from oxidative stress and ferroptosis (20). Since mutations in BRAF and NRAS comprehensively account for about 70% of melanoma cases, it is tempting to suggest that activation of RAS/RAF/MAPK pathway may as well influence the susceptibility of this tumor type to ferroptosis.

Even though, the lack of functional studies on ferroptosis using large cohorts of tumors with known RAS mutational status prevents to go beyond mere speculations, the DepMap effort (https://depmap.org/portal/) offers a resourceful tool to make hypothesis and correlations based on cell line screening data (21–23).

**Figure 1:**
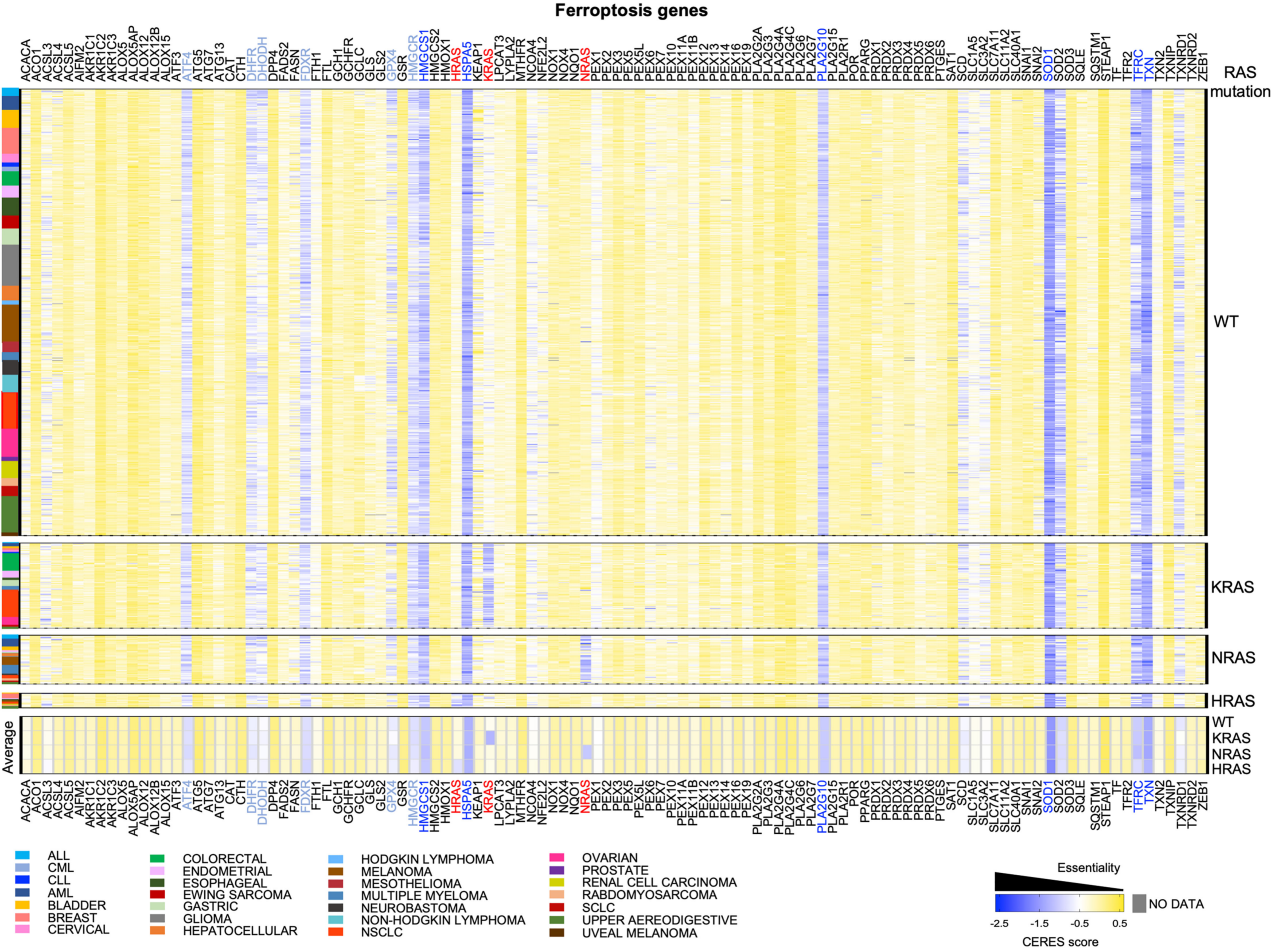
CRISPR knock-out screening from DepMap to identify dependencies on ferroptosis genes in RAS-WT and RAS-MUT cancer cells. The heatmap shows the median CERES dependency (DepMap_21Q3_Public,_CERES) from https://depmap.org/portal/ for the indicated ferroptosis genes (x axes) in the indicated type of cancer cell lines (y axis). The CERES score defines whether a given human cancer cell line is dependent on a given gene: a gene with CERES score of 0 or greater is considered non-essential, whereas a score lower than -0.5 indicates a likely dependency, with -1 indicating all common essential genes. Median CERES score was calculated for each lineage_2 parameter reported in the color legend. Cells were grouped according to the absence (RAS-WT) or the presence of mutations in of *KRAS, NRAS, HRAS* mutations. The bottom part of the heatmap displays the median CERES score for all the cancer cells of the indicated genotype. *RAS* genes are indicated in red, genes having an overall median CERES score of -1 or lower are indicated in dark blue and genes with a score between -0.8 and -0.9 are reported in light blue.

Here, we took advantage of the whole-genome CRISPR knock-out screening to analyze whether, several genes involved in ferroptosis are differentially required in tumor cells having mutations in the RAS genes (KRAS, NRAS and HRAS) and those harboring KRAS-WT. First of all, what comes obvious looking at the heatmap in **Figure 1**, is that each RAS gene is essential in the relative cohort of tumor cells harboring activating mutations in that specific RAS gene. This might represent a “positive” control in our analysis, indicating that the driver RAS mutations impose an actionable dependency in that specific cohort. Accordingly, doxycycline (dox)-inducible KM mice have shown that KM is necessary for tumorigenesis and that extinction of KM leads to complete tumor regression (24, 25). This observation triggered the development of KRAS inhibitors that are now FDA-approved (26–30). However, functional studies are revealing that the success of KM inhibitors is challenged by drug resistance, as it happens for the majority of cancer therapies (31, 32). This problem might be due to tumor heterogeneity within the bulk tumor: some tumor cells may be intrinsically independent of KM and others may become independent of KM overtime, upon adaptation to stress (therapy, metabolism, crosstalk with microenvironment, redox balance).

These aspects apply to virtually all genes, especially those involved in ferroptosis, which is indeed intertwined with metabolism and oxidative stress. DepMap screening does not take those variables into consideration, therefore, here, we merged DepMap data with the existing literature looking for commonalities, discrepancies, and potential new insights on ferroptosis.

## Potential Pan-Cancer Ferroptosis Vulnerabilities

**Figure 2:**
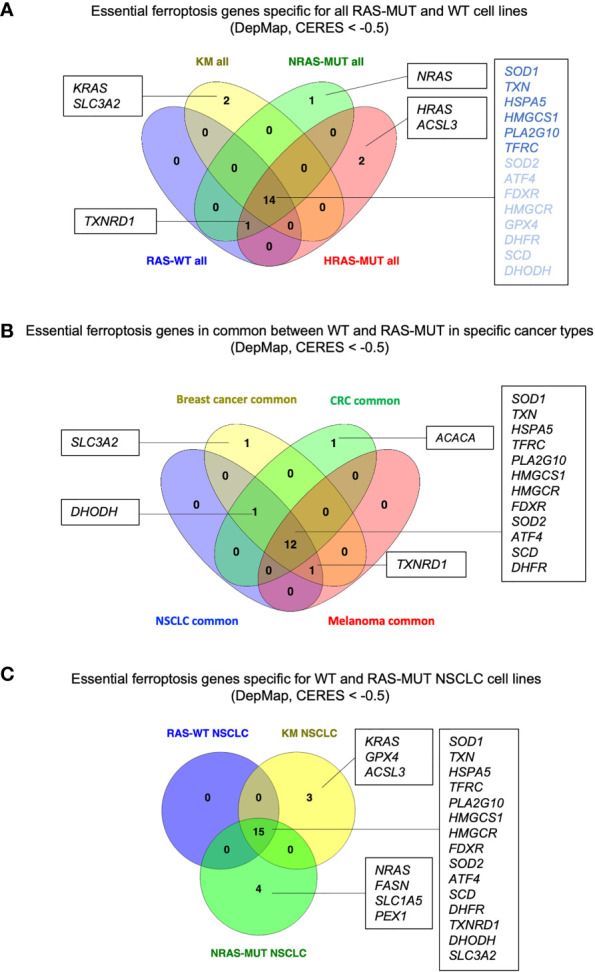
RAS- and cancer type-independent/dependent ferroptosis vulnerabilities. **(A)** Venn diagram summarizing the genes with CERES scores lower than -0.5 in cancer lines grouped for RAS mutation. RAS-independent genes with overall median CERES lower than -1 are indicated in dark blue. **(B)** Venn diagram for the RAS-independent genes with CERES scores lower that -0.5 in breast cancer, colorectal cancer (CRC), non-small cell lung cancer (NSCLC) and melanoma cell lines. **(C)** Venn diagram showing the ferroptosis genes with CERES score lower than -0.5 in NSCLC cell lines of the indicated genotype. Diagrams created using Venny 2.1 (86).

Among the ferroptosis genes that we took into consideration, it is worth to note that some of them are potential pan-essential genes in all the cancer cell lines, independently of RAS mutational status and of the cancer type. Those can be seen as blue lines running across the heatmaps in **Figure 1** and summarized in the venn diagrams reported in **Figures 2A, B** (CERES score <-0.5). They include the Activating Transcription Factor 4 (*ATF4*), Heat Shock Protein Family A Member 5 (*HSPA5)*, dihydrofolate reductase (*DHFR*), ferredoxin reductase (*FDXR*), glutathione peroxidase 4 (*GPX4*), 3-Hydroxy-3-Methylglutaryl-CoA Reductase (*HMGCR*), 3-Hydroxy-3-Methylglutaryl-CoA Synthase 1 (*HMGCS1*), Phospholipase A2 Group X (*PLA2G10*), superoxide dismutase 1 and 2 (*SOD1* and *SOD2*), transferrin receptor (*TFRC*) and thioredoxin (*TXN*). Among them, *SOD1, TXN, HSPA5, HMGCS1, PLA2G10* and *TFRC* are the top pan-essential ferroptosis-related genes, having an average CERES score lower than -1.

Which is the best pan-cancer candidate gene involved in ferroptosis?

SOD1 actively participates in the reactive oxygen species (ROS) detoxification converting the superoxide radicals to molecular oxygen and hydrogen peroxide. Therefore, it’s no surprise to find it among the top lethal ferroptosis genes. However, recent findings have reported a novel pro-tumorigenic role of SOD1 specifically in KMLC, where it promotes ribosomal biogenesis in the nucleus (33). Also, activating mutation of SOD1 have been described to promote instead of inhibiting ferroptosis in Amyotrophic Lateral Sclerosis (ALS) (34). Therefore, additional studies about SOD1 in cancer and ferroptosis are needed to establish its role and whether it might be used as a pan-cancer targetable dependency.

*TXN* encodes for thioredoxin, a thiol-dependent antioxidant protein able to reduce oxidized cysteines on target proteins, including the conversion of cystine into cysteine. In this context, inhibition of TXN using ferroptocide has been shown to induce ferroptosis in several cancer cells, indicating that TXN might be *a bone fide* pan-cancer dependency protecting from ferroptosis (35).

*HSPA5* encodes for a chaperone which participates to the unfolded protein response promoting cell survival under conditions of ER stress (36). Recently, HSPA5 has been found to directly prevent ferroptosis by impeding GPX4 degradation in KM pancreatic ductal adenocarcinoma (PDAC) (37, 38) and in glioma cells (39). These studies are in agreement with previous work from our lab, showing that unresolved ER-stress hinders tumorigenesis in KMLC and that synthetic chaperone treatment can reverse this phenotype (40). Additional functional studies including other cancer types will elucidate whether *HSPA5* is a pan-essential ferroptosis gene or specific for RAS-driven malignancies under stress.

*HMGCS1* encodes for the enzyme catalyzing the synthesis of HMG-CoA, the first step of the mevalonate pathway. This pathway gives rise to isopentenyl pyrophosphate (IPP) which is not only the precursor of squalene, cholesterol and CoQ10, but also the essential substrate for the isopentenylation of Sec-tRNA, a process necessary for the synthesis of selenoproteins, including GPX4. All the downstream products of the mevalonate pathway have implications in ferroptosis prevention and inhibition of this pathway has been proven to be an effective way to induce ferroptosis in several models (17, 41–43).

*PLA2G10* encodes for a secretory calcium-dependent phospholipase A2 that primarily targets extracellular phospholipids (PL). This enzyme remodels circulating phosphatidylcholines releasing lysophosphatidylcholines and PUFA (44, 45), which are substrates for lipid peroxidation (2, 11, 46). It is reasonable to think that this secretory PLA2 can counteract the availability of PUFA-containing PL, thereby preventing cancer cells from ferroptosis. This is in line with our study showing that PLA2G4C, a cytosolic PLA2, is required to prevent ferroptosis in KMLC upon FASN inhibition (11).

*TFRC* encodes for the transferrin receptor that mediates cellular uptake of iron-loaded transferrin (TF). Following binding to TFRC, the TF-TFRC complex is internalized *via* receptor-mediated endocytosis. In the endosome (an acidic environment), the released iron needs to be reduced from Fe3+ to Fe2+ by transmembrane reductases of the STEAP family. Noteworthy, TRFC is involved in iron uptake and has been described as a pro-ferroptosis marker (47). Accordingly, expression of *TFRC* has been reported to positively correlate with sensitivity to ferroptosis inducers (5, 48), while *TFRC* knock-out protected RAS-driven cancer cells from erastin-induced ferroptosis (5). Additionally, recent studies have shown that iron accumulation in KRAS-driven tumors can be exploited to selectively target cancer cells with iron-activatable drugs (49). In this context, it is quite surprising to observe that in the DepMap data *TFRC* is instead a pan-cancer lethality. This seemingly counterintuitive results might be explained by the broader role that TFRC and iron metabolism have in cell biology (50, 51). Therefore, additional functional studies are necessary to clarify whether TFRC is a pro-ferroptosis marker or, perhaps, it is a pan-cancer dependency independently of ferroptosis.

## Are There Cancer Type-Specific Ferroptosis Dependencies?

We took into consideration the essential ferroptosis genes that are independent of RAS mutation in non-small cell lung cancer (NSCLC), breast cancer, colorectal cancer (CRC) and melanoma, which are among the leading causes of cancer-related deaths. Even though the pan-cancer genes remain the same discussed in the section above, **Figure 2B** suggests some potential cancer-specific vulnerabilities. For instance, breast cancer cell lines seem to depend on *SLC3A2* which encodes for the heavy chain subunit of the cystine-glutamate antiporter (xCT system), along with SLC7A11. These data are in agreement with studies indicating that SLC3A2 predicts poor prognosis in breast cancer and xCT expression dictates ferroptosis sensitivity in breast cancer cells (52, 53). However, it is worth to note that while SLC7A11 has been recognized to be the key player in ferroptosis, SLC3A2 has been usually reported more as a chaperon protein supporting SLC7A11 stability and localization (54). Therefore, it is quite surprising to see that *SLC3A2* is essential while *SLC7A11* is not. Perhaps, this suggests that SLC3A2 may have additional roles in tumorigenesis that are ferroptosis independent. Indeed, SLC3A2 can both couple with other amino acid transporters and mediate cell-matrix crosstalk during tumorigenesis (55, 56). More importantly, a recent study has demonstrated that IgE monoclonal antibody and CAR T cell immunotherapies recognizing SLC3A2 can successfully hinder cancer growth without overt systemic toxicities (57). Future studies will help elucidate whether SCL3A2 is a specific vulnerability of breast cancer and its role in ferroptosis.

Our analysis also suggests that ACACA may be a specific vulnerability of CRC. *ACACA* encodes for ACC1, the enzyme that catalyzes the conversion of acetyl-CoA to malonyl-CoA, the rate-limiting step in fatty acid synthesis. ACC1 has been reported to have pro-tumorigenic role in many cancers (58, 59) as well as to have tumor suppressor roles in other tumors, such as hepatocellular carcinoma (60). In addition, ACC1 has been reported to modulate the sensitivity to ferroptosis inducers in a cell context-dependent manner (61). However, little is known about its role in CRC. Therefore, it may be useful to study whether ACC1 and lipid metabolism play a role in CRC tumorigenesis and progression, and how its functions can be linked to ferroptosis in this disease.

Another insight coming from **Figure 2B** is that Dihydroorotate Dehydrogenase (DHODH) seems to be a common vulnerability in CRC, NSCLC and breast cancer, irrespectively of the RAS mutational status. DHODH is a mitochondrial enzyme which catalyzes a rate limiting step of the *de novo* pyrimidine synthesis pathway, converting dihydroorotate (DHO) to orotate. Inhibition of DHODH has been recently proposed to be effective against acute myeloid leukemia (AML), breast and colorectal cancers, and to induce ferroptosis in several cancer cell lines and patient-derived xenografts (19, 62, 63). Several clinical trials are currently evaluating DHODH inhibitors as anti-cancers (63) and will elucidate whether DHODH is a specific vulnerability of CRC, NSCLC and breast cancer.

Finally, NSCLC, breast cancer and melanoma share dependency on *TXNRD1* which encodes for the selenoprotein thioredoxin reductase. TXNRD1 along with gluthathione (GSH) generates the intracellular pool of cysteine by reducing the cystine uptaken *via* xCT. Upon GSH depletion, TXNRD1 can maintain the intracellular pool of cysteine and GPX4 sustaining cell survival (64, 65). Accordingly, a study showed that auranofin, an irreversible inhibitor of TXNRD1, in combination with GSH depletion can induce ferroptosis in small cell lung cancer (SCLC) (18). However, other studies have suggested that silencing of *TXNRD1* promote resistance to ferroptosis inducers in cancer cells (66). Previous work has shown that novel gold compounds targeting TXNRD1 are able to induce cell death in breast and lung cancer cell lines (67, 68). It will be of interest to further develop pharmacolocical tools targeting TXNRD1 in order to understand whether it can be harnessed to induce ferroptosis.

## RAS-Mutant Specific Ferroptosis Vulnerabilities in NSCLC

Within the same tumor type, are there ferroptosis vulnerabilities specific for a given RAS-mutation?

For instance, we took into consideration NSCLC cell lines which harbor RAS-WT or mutations in either KRAS (KM) or NRAS (NRAS-MUT) genes (**Figure 2C**). It is worth to note that while the common, RAS-independent genes remained the same described previously, KM and NRAS-MUT LC cell lines exhibit specific and different ferroptosis gene candidates. Indeed, in KMLC cells along with the gene *KRAS* itself, also *GPX4* and Acyl-CoA Synthetase Long Chain Family Member 3 (*ACSL3*) appear as potential dependencies. GPX4 is one of the best-known enzymes protecting from ferroptosis and its deletion produces uncontrolled LPO and cells death (6, 69). As it regards KMLC, there is evidence that GPX4 inhibition can not only re-sensitize cells to radiotherapy (70), but it can also synergize with the inhibition of other pathways, such as NFR2 (71) and mTORC1 (72).

In addition, several cancer therapies used in KMLC have been reported to directly or indirectly affect GPX4 (73). Therefore, using GPX4 inhibition in combination with chemotherapy/radiotherapy may represent a valuable strategy to induce ferroptosis in KMLC.

However, as several studies suggested, not all the cancer cells exhibit the same requirement for the cysteine/GSH/GPX4 pathway, and they can escape ferroptosis through other ways.

As it regards ACSL3, our lab has previously reported that it is a selective vulnerability of KMLC (74). Indeed, ablation of ACSL3 impairs *in vivo* tumorigenesis in KMLC mice by disrupting lipid beta-oxidation and fat composition of cancer cells (74). Indeed, ACSL3 is one of the enzymes able to conjugate fatty acids (FAs) to coenzyme A, which is essential for FAs to be utilized/metabolized. Interestingly, other studies have revealed that ACSL3 is also necessary to protect cancer cells from ferroptosis, by promoting the incorporation of SFA and MUFA (20, 75). Also, ACSL3 has been described to play a role in PUFA metabolism and the synthesis of PUFA-derived signaling molecules in NSCLC (76). This evidence is in agreement with KMLC having a dependency on *de novo* lipogenesis and the remodeling of FAs to escape ferroptosis (11).

Future and ongoing efforts in our lab are focused on understanding whether and how ACSL3 protects KMLC cells from ferroptosis in preclinical models and how it reshapes the lipid composition in this type of cancer.

NRAS-MUT NSCLC cell lines, on the other hand, exhibit a specific dependency on *NRAS* itself, *FASN*, Solute Carrier Family 1 Member 5 (*SLC1A5*) and Peroxisomal Biogenesis Factor 1 (*PEX1*).

FASN, the rate-limiting enzyme for fatty acid synthesis, has been already shown to be a dependency of KM and NRAS-MUT NSCLC (11, 77).

However, unlike KMLC, there are no studies demonstrating that inhibition of FASN induces ferroptosis NRAS-MUT NSCLC. Several novel FASN inhibitors are being tested in clinical trials and it will be of interest to establish whether FASN might be targeted to induce ferroptosis in NRAS-MUT NSCLC.

*SLC1A5* encodes for a sodium-dependent transporter of neutral amino acid, especially glutamine, which directly contributes to ferroptosis (78). Some recent works indicated that SLC1A5 might have a dual role in cancer. Indeed, if high expression of *SLC1A5* corelates with poor prognosis in pancreatic adenocarcinoma, it seems also to predispose melanoma cells to ferroptosis (79, 80). It may be of interest to survey whether NRAS-MUT LC depends on SLC1A5 to escape ferroptosis or rather to satisfy glutamine requirements during proliferation.

The other gene that seems to be specific for NRAS-MUT LC is *PEX1* which encodes for a large ATPase involved in peroxisome biogenesis. Interestingly, *PEX1* and other *PEX* genes have been proposed as pro-ferroptosis genes (81). Indeed, by sustaining peroxisome biogenesis and function, they promote the synthesis of polyunsaturated ether phospholipids (PUFA-ePLs), which in turn cause ferroptosis (81). However, this study is in contrast with DepMap data showing that *PEX1* loss is detrimental to NRAS-MUT LC cells and with other work showing that ether ePLs are actually acting as antioxidants (82). As suggested by Aldrovandi and Conrad in their research highlight, this apparent contradictory role of ePLs might be due to the diverse degree of unsaturation, chain length of esterified FAs and the composition of surrounding lipids (83). This hypothesis is in agreement with other studies showing that the quality of PL and ePLs, rather than the quantity, makes the difference in terms of ferroptosis susceptibility (11, 84, 85).

Future studies about the lipid composition of NRAS-MUT LC cells may help uncovering potential roles of ePLs and peroxisomes in this type of cancer.

## Conclusions

The purpose of this perspective was to determine potential relationships between ferroptosis-related genes and RAS oncogenes across several cancer types.

Taking advantage of the DepMap screening tool, we classified several genes as potential pan-cancer vulnerabilities, disregarding the RAS mutational status. On the other hand, some other genes seem to be cell-context and RAS-mutation dependent. This evidence is potentially very useful because it suggests that general and tumor-specific pathways might be exploited to induce ferroptosis in different cancers.

Also, dependencies that seem to correlate with RAS-mutational status, may be investigated along with current chemotherapies to potentiate anti-tumor effects. However, these data suffer from obvious limitations: as previously said in this perspective, DepMap data do not consider the metabolic dynamics, the plasticity and the stress factors influencing tumor susceptibility to ferroptosis.

Furthermore, it is compelling to note that the use of preclinical models of RAS-driven cancers is massive in the study of ferroptosis. Thus, a lingering question remains: is this because these models are well-established and widespread in cancer biology or is it because they have enhanced sensitivity to ferroptosis? Therefore, in the future, it will be of interest to compare DepMap data with functional studies aimed at better dissecting how the ferroptosis genes herein discussed affect RAS-driven cancers. The take-home message is that there is no single flavor of ferroptosis, and genes involved in ferroptosis exert also other functions in different cell-contexts. RAS-driven cancers are no exception to this paradigm and additional evidence is required to distinguish correlations from causations.

## Data Availability Statement

The data analyzed in this perspective are available at https://depmap.org/portal/. Further inquiries can be directed to the corresponding author.

## Author Contributions

CA and CB performed literature and data analysis, organized and critically reviewed available information and conceived how to structure the review. They also wrote the manuscript and made the figures. PS supervised the work and critically revised the manuscript. All authors contributed to the article and approved the submitted version.

## Conflict of Interest

The authors declare that the research was conducted in the absence of any commercial or financial relationships that could be construed as a potential conflict of interest.

## Publisher’s Note

All claims expressed in this article are solely those of the authors and do not necessarily represent those of their affiliated organizations, or those of the publisher, the editors and the reviewers. Any product that may be evaluated in this article, or claim that may be made by its manufacturer, is not guaranteed or endorsed by the publisher.
